# Impact of anesthesia management during cytoreductive surgery plus hyperthermic intraperitoneal chemotherapy for the treatment of colorectal peritoneal carcinomatosis on intra- and postoperative outcomes: A systematic review protocol

**DOI:** 10.1097/MD.0000000000016467

**Published:** 2019-07-26

**Authors:** Moulay Idrissi, Fabien Espitalier, Richard Coveney, Marie-Eve Bélanger, Quentin Gobert, Lucas Sidéris, Pierre Dubé, Olivier Verdonck, Louis-Philippe Fortier, Philippe Richebé

**Affiliations:** a Maisonneuve-Rosemont Hospital Research Center (CR-HMR); b Department of Anesthesiology and Pain Medicine; c Department of Oncology Surgery, Maisonneuve-Rosemont Hospital, CIUSSS de l’Est-de-l’Île-de-Montréal, School of Medicine, University of Montreal; d Direction of University Education of CIUSSS de l’Est-de-l’Île-de-Montréal; e Maisonneuve-Rosemont Hospital Library, Montreal, QC, Canada.

**Keywords:** colorectal peritoneal carcinomatosis, cytoreductive surgery, hyperthermic intraperitoneal chemotherapy

## Abstract

**Background:**

The impact of the anesthesia management during cytoreductive surgery (CRS) plus hyperthermic intraperitoneal chemotherapy (HIPEC) for the treatment of colorectal peritoneal carcinomatosis (CRPC) on postoperative outcomes might be of major importance in the process of postoperative recovery. It might have a significant impact on intra- and postoperative outcomes, but the evaluation of this impact seems to be under-reported. To investigate the question whether the anesthesia management was reported in previous studies done in this population and if it had any impact on postoperative outcomes, we propose to conduct a systematic review of the published literature.

**Methods:**

For this review, we will follow the Preferred Reporting Items for Systematic Reviews and Meta-Analyses Protocols (PRISMA-P). Medline/PubMed, Embase, EBM Reviews and the Cochrane Database of Systematic Reviews (CDSR) will be systematically consulted for eligible studies without age, gender, ethnic, and language restriction. The goal of this review will be to assess whether anesthesia monitoring, dosing, and analgesia protocols were reported in this literature on this specific procedure and whether the impact of the anesthesia management on intraoperative safety and postoperative recovery was evaluated.

**Results:**

The results of this systematic review will allow to answer the initial question: has the impact of anesthesia management on intraoperative safety and patients’ postoperative recovery already been studied and reported in the past for this type of major surgery? And does anesthesia have any impact on postoperative outcomes?

**Discussion:**

In the hypothesis that the impact of anesthesia management on patients’ postoperative recovery has never been studied, or very little reported in previous studies in this type of major surgery, it would be justified to conduct a randomized controlled trial on this specific objective.

**Registration:**

This systematic review protocol was registered in PROSPERO, under the registration number CRD42019124162.

## 1 Introduction

Colorectal cancer (CRC) is the third most common tumor world-wide.^[1,2]^ This is a major health problem with a world-wide incidence of 1.4 million in 2012.^[3,4]^ Colorectal peritoneal carcinomatosis (CRPC) is a common clinical phenomenon in patients with colorectal cancer and has been reported to have a poor prognosis.^[5]^ The development of a novel treatment approach combined aggressive cytoreductive surgery (CRS) to remove all visible tumors, hyperthermic intraperitoneal chemotherapy (HIPEC) to eradicate microscopic residual disease^[6]^ and intraoperative intravenous chemotherapy.^[7–9]^ This new comprehensive treatment improves the median overall survival (OS) of selected patients with CRPC up to 21 to 63 months, and 5-year survival rate up to approximately 40%,^[10,11]^ or even 58% according to the American Society of Peritoneal Surface Malignancies (ASPSM) multi-institution study.^[12]^ It has been widely recognized in North America, Europe, Australia, and Japan.^[5,13–16]^ In the 9th International Congress on Peritoneal Surface Malignancies in Amsterdam in 2014, peritoneal surface oncology group international (PSOGI) reached a consensus that CRS+HIPEC should be considered as the standard therapy for the selected patients with mild to moderate CRPC.^[17]^

Nevertheless, we think that study of this comprehensive treatment strategy for CRPC patient remains incomplete due to lack of information on the impact of strategy adopted by the anesthesiology team during surgery. Therefore, we will perform a systematic review of published clinical studies to verify whether or not the strategy adopted by the anesthesiology team was mentioned and considered by surgeons in their evaluations and publications.

## 2 Methods and analysis

The protocol of this systematic review is based on Preferred Reporting Items for Systematic Reviews and Meta-Analyses Protocols (PRISMA-P) checklist,^[18,19]^ as well as PRISMA guidelines.^[20]^ The systematic review will be conducted following an established protocol and recorded in PROSPERO, under reference: CRD42019124162. This study is a literature analysis which does not involve human subjects and therefore does not require the ethics committee approval.

### 2.1 Eligibility criteria

Selection criteria are defined using PICOS approach (Patients, Intervention, Comparison, Outcomes, and Study design type). In this approach study, we want to know if during CRS + HIPEC (P) surgeries, a monitored and optimized general anesthesia (I) versus a conventional general anesthesia (C) could reduce the length of hospital stay of patients (O). We will include all studies that contain patients with colorectal cancer treated by Cytoreductive surgery (CRS) plus hyperthermic intraperitoneal chemotherapy (HIPEC), without age, gender, ethnic and language restriction. We will exclude all peritoneal carcinomatosis that is not of colorectal origin, for example, peritoneal carcinomatosis of ovarian or appendicular origin.

### 2.2 Information sources

To do this systematic review, we will explore 4 separate databases. Medline/PubMed, Embase, EBM Reviews and the Cochrane Database of Systematic Reviews (CDSR) will be systematically consulted for eligible randomized controlled trials without age, gender, ethnic and language restriction. The previous databases will be searched by a professional study librarian and reviewed by 2 researchers.

### 2.3 Search strategy

The research strategy is to make a first search by MeSh [MH], complete with a second research by keywords in titles and abstracts [tiab], according to the following procedure.

#1: “cytoreduction surgical procedures”[MH]

#2: “Hyperthermia, Induced”[MH]

#3: “colorectal neoplasms”[MH]

#4: oxaliplatin[NM]

#5: #1 AND #2 AND #3 AND #4

“cytoreduction surgical procedures”[MH] AND “Hyperthermia, Induced”[MH] AND “colorectal neoplasms”[MH] AND oxaliplatin[NM]

#6: (cytoreduction[tiab] OR cytoreductive[tiab] OR debulking[tiab])

#7: (hyperthermia[tiab] OR hyperthermic[tiab] OR HIPEC[tiab])

#8: colorectal[tiab]

#9: oxaliplatin[tiab]

#10: #6 AND #7 AND #8 AND #9

(cytoreduction[tiab] OR cytoreductive[tiab] OR debulking[tiab]) AND (hyperthermia[tiab] OR hyperthermic[tiab] OR HIPEC[tiab]) AND colorectal[tiab] AND oxaliplatin[tiab].

### 2.4 Study records

**Figure 1:**
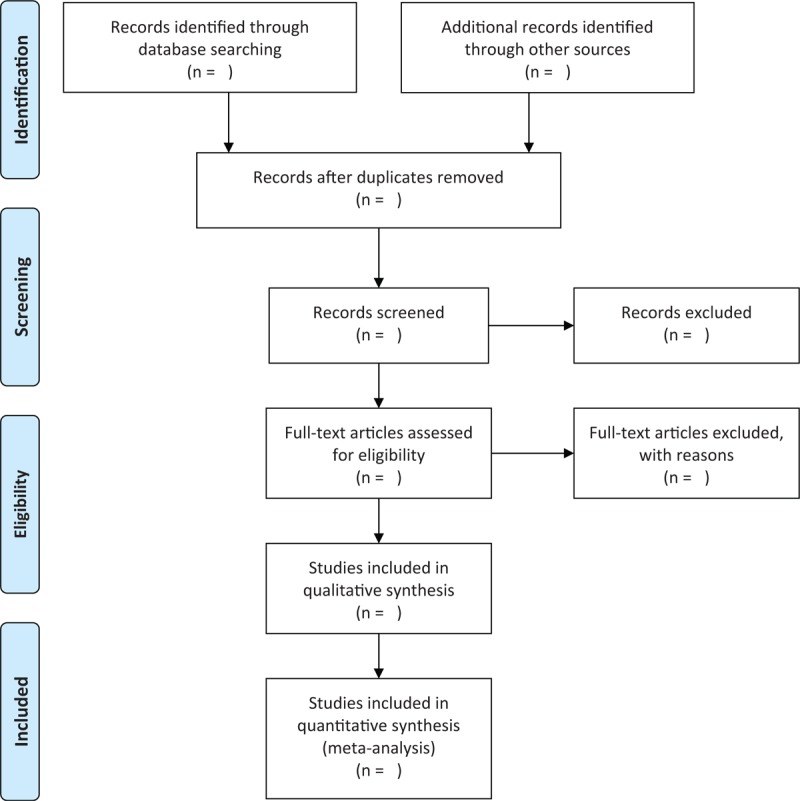
PRISMA flow diagram. Adapted from: Moher D, Liberati A, Tetzlaff J, Altman DG, The PRISMA Group (2009). Preferred Reporting Items for Systematic Reviews and Meta-Analyses: The PRISMA Statement. PLoS Med 6(7): e1000097. doi:10.1371/journal.pmed1000097.

All search results from different databases will be exported to the Rayyan QCRI (Qatar Computing Research Institute, Data Analytics Medical) web application for analysis (Rayyan QCRI is a web/mobile application to help systematic review authors screen citations in a quick, easy and enjoyable fashion). We will use Rayyan QCRI web application to manage records and data throughout the review. We will start by eliminating all the duplicate records with this application, before starting the blind study selection process by both examiners. Titles and abstracts will be systematically selected for eligibility by 2 independent researchers. Disagreements will be resolved by consensus between the 2 study investigators. The selection process of the articles will be summarized in a PRISMA flow diagram (Fig. 1).

At the end of the selection, the selected studies will be exported and listed in EndNote. The full texts of the selected studies will be rigorously studied by 2 researchers to extract all the relevant information we need to answer our initial question.

### 2.5 Data items and outcomes

The objective of this review will be to assess in the selected studies whether the impact of the anesthesia management on intraoperative safety and postoperative recovery in patients undergoing cytoreductive surgery (CRS) plus hyperthermic intraperitoneal chemotherapy (HIPEC) for the treatment of colorectal peritoneal carcinomatosis (CRPC) has been reported. To do so, we will evaluate whether the authors described in their methods or results the intraoperative anesthesia monitoring (depth of anesthesia, entropy, BIS) and dosing (absorption and consumption of anesthetic gases), the intraoperative analgesia monitoring (depth of analgesia, pupillometry, PPI, ANI, NOL, cutaneous conductance) and the intraoperative hymodynamic monitoring (arterial cannula, cardiac output, delta pp) and their impact on length of hospital stay.

### 2.6 Risk of bias individual studies

In order to avoid the risk of bias in individual studies, each included article will be independently evaluated by 2 separated investigators. There are different assessment tools that can be used depending on each study design. We will use the Cochrane Collaboration's tool for assessing risk of bias in all Randomized Controlled Trials.^[21]^ The risk of bias evaluation will be done at the study level. At the end of the study, disagreements concerning the risk of bias evaluation between the 2 investigators will be resolved in group discussion to achieve consensus.

### 2.7 Data synthesis

Results of the study selection process will be described and summarized using a PRISMA Flowchart [Fig. 1]. Once the articles have been identified, the duplicates will be eliminated. The remaining articles will be screened first on their title and/or on their abstract and/or on the body of the article. All selected articles will then be evaluated in their full text. The data of interest will then be extracted. We will perform a qualitative description of the studies included, and we will summarize and synthesize the data in a narrative approach.

### 2.8 Meta-biases

All potential meta-biases will be evaluated in each selected study, such as publication bias across studies, selective reporting within studies.

### 2.9 Confidence in cumulative evidence

We want to do a quantitative analysis of the literature based on our predefined selection criteria. In the event that the available data are insufficient or too heterogeneous, we will perform a qualitative analysis of the literature by giving a narrative evaluation of publications describing anesthesia in HIPEC surgeries. We will also give the percentage of publications that took into account the impact of anesthesia in their study and their conclusions.

## 3 Discussion

Currently, the evaluation of the impact of anesthesia during Cytoreductive surgery (CRS) plus hyperthermic intraperitoneal chemotherapy (HIPEC) in the treatment of colorectal peritoneal carcinomatosis (CRPC) is still poorly reported. This is why we will conduct a literature systematic review to investigate on this question. To our knowledge, this will be the first systematic review that will evaluate how much anesthesia details are provided in this surgical literature and what is the anesthesia impact on postoperative outcomes in this type of complex procedure. We assume this review will highlight the lack of information concerning anesthesia in most of the publications available to date in this major surgery, and thus the need of future studies to answer this specific question.

## Author contributions

**Conceptualization:** Moulay Idrissi, Philippe Richebé.

**Data curation:** Moulay Idrissi, Fabien Espitalier, Richard Coveney.

**Formal analysis:** Moulay Idrissi.

**Funding acquisition:** Philippe Richebé.

**Investigation:** Moulay Idrissi, Fabien Espitalier, Richard Coveney.

**Methodology:** Moulay Idrissi, Philippe Richebé.

**Project administration:** Moulay Idrissi, Philippe Richebé.

**Resources:** Richard Coveney.

**Supervision:** Philippe Richebé.

**Validation:** Fabien Espitalier, Marie-Eve Bélanger, Quentin Gobert, Lucas Sidéris, Pierre Dubé, Olivier Verdonck, Louis-Philippe Fortier, Philippe Richebé.

**Visualization:** Moulay Idrissi.

**Writing – original draft:** Moulay Idrissi.

**Writing – review & editing:** Fabien Espitalier, Marie-Eve Bélanger, Quentin Gobert, Lucas Sidéris, Pierre Dubé, Olivier Verdonck, Louis-Philippe Fortier, Philippe Richebé.
